# Crystal structures of 1-bromo-3,5-bis­(4,4-dimethyl-1,3-oxazolin-2-yl)benzene 0.15-hydrate and 3,5-bis­(4,4-dimethyl-1,3-oxazolin-2-yl)-1-iodo­benzene

**DOI:** 10.1107/S2056989015016059

**Published:** 2015-09-12

**Authors:** Timo Stein, Frank Hoffmann, Michael Fröba

**Affiliations:** a Institute of Inorganic and Applied Chemistry, Department of Chemistry, University of Hamburg, Martin-Luther-King-Platz 6, D-20146 Hamburg, Germany

**Keywords:** crystal structure, 2-oxazolines, bromoar­yl, iodoar­yl, Phebox ligands, hydrogen bonding, parallel-displaced π–π inter­action, N⋯I contacts, isostructural compounds

## Abstract

The bromo and iodo derivatives of a *meta*-bis­(1,3-oxazolin-2-yl)-substituted benzene, C_16_H_19_BrN_2_O_2_·0.15H_2_O (**1**) and C_16_H_19_IN_2_O_2_ (**2**), have been prepared and studied in terms of their mol­ecular and crystal structures. While the former crystallizes as a sub-hydrate, with 0.15 formula units of water and shows an almost all-planar arrangement of the three ring systems, the latter crystallizes solvate-free with the flanking heterocycles twisted considerably with respect to the central arene. Non-covalent contacts include parallel-displaced π–π inter­actions and (non-classical) hydrogen bonding for both (**1**) and (**2**), as well as relatively short I⋯N contacts for (**2**).

## Abstract 2

The isostructural 1-bromo and 1-iodo derivatives of 3,5-bis­(1,3-oxazolin-2-yl)benzene show supra­molecular features of (non-classical) hydrogen bonding, parallel-displaced π–π inter­actions, and close N⋯I contacts. The former was found to crystallize as a sub-hydrate.

## Chemical context   

The 2-oxazolinyl functional group has been employed as a protective group for carb­oxy­lic acids rendering them stable against organometallic reagents (Wuts & Greene, 2007 ▸). Aromatic 1,3-substituted bis­(1,3-oxazolin-2-yl) compounds have shown to be efficient for directed *ortho* metallation (DoM) reactions (Harris *et al.*, 1978 ▸), while competitive halogen–metal exchange reactions should be considered for the halide-substituted title compounds reported herein. The substitutional pattern also gives access to bis­(1,3-oxazolin-2-yl) systems suitable for the preparation of *N*,*C*,*N*-tridentate pincer ligands which have come to general attention as Phebox ligands [Phebox: 2,6-bis­(1,3-oxazolin-2-yl)phen­yl].

There are many examples for respective organometallic complexes of transition and rare-earth metals known in the literature with many of them bearing chiral 2-oxazolinyl substituents within the Phebox ligand. Exemplary compounds include those of some early transition metals (Chuchuryukin *et al.*, 2011 ▸), iridium (Allen *et al.*, 2014 ▸), or palladium (Lu *et al.*, 2010 ▸), to name but a few.

The substitution with bromine and iodine renders the title compounds capable of being utilized as potential precursors for C–C cross-coupling building blocks bearing the Phebox motif.
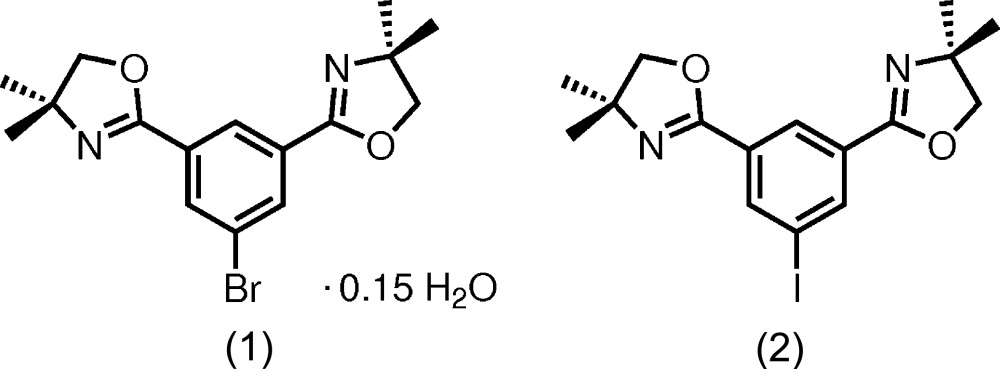


## Structural commentary   

The structural considerations below take least-squares mean planes for rings *A* (O1/C7/N1/C9/C8) and *C* (O2/C12/N2/C14/C13) of the heterocyclic 2-oxazolinyl groups and *B* (C1/C2/C3/C4/C5/C6) for the central arene into account. For both (**1**) and (**2**), the 2-oxazoline moiety with an approximately accordant orientation of the C=N bond with respect to the C5—*X*1 (*X* = Br, I) bond was chosen to be denoted as ring *A*.

**Figure 1:**
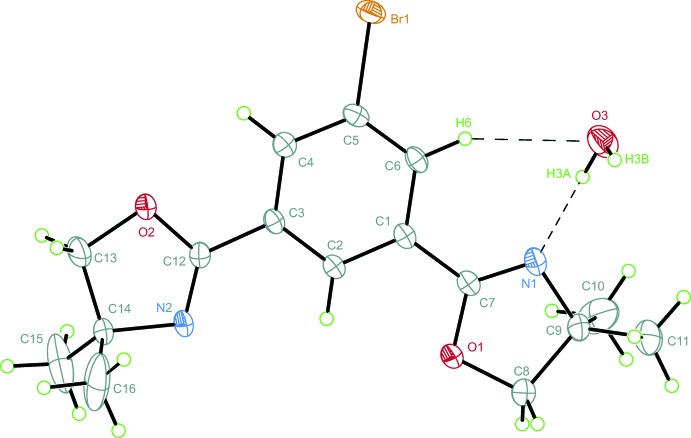
The asymmetric unit of (**1**), shown with 50% probability displacement ellipsoids. H atoms are drawn as green spheres of an arbitrary radius.

Compound (**1**) crystallized from water-containing aceto­nitrile as an adduct with 0.15 H_2_O in the monoclinic space group *P*2_1_/*n*. Within the mol­ecular structure (Fig. 1 ▸) the 2-oxazolinyl functional groups are oriented anti­periplanar to each other. The elongated displacement ellipsoid of the methyl­ene carbon atom C13 indicates a noteworthy vibrational freedom orthogonal to plane *C* which was not observed for atoms within ring *A*. The absence of a comparable displacement component of C14 perpendicular to plane *C* disagrees with pseudorotational disorder around the atomic positions mentioned.

The five-membered heterocyclic moieties possess different conformational characteristics. Within ring *A*, significant puckering with parameters *τ_m_* = 13.2 (1)°, *q*_2_ = 0.1284 (19) Å, and *φ*_2_ = 134.2 (9)° indicate a ^C8^ *T*_C9_ conformation distorted towards ^C8^ *E* (Altona & Sundaralingam, 1972 ▸; Cremer & Pople, 1975 ▸). Ring *C* shows only slight deviation from ideal planarity with *τ_m_* = 3.5 (1)°.

Inter­planar angles of 2.15 (12)° and 3.67 (16)° of the planes N1/C7/O1 and N2/C12/O2 with plane *B*, respectively, have been found for the almost all-planar overall structure. Considerable angular deviations from ideal (120°) angles within ring *B* were found for C4—C5—C6 = 122.04 (15)°, C3—C4—C5 = 118.75 (15), and C1—C6—C5 = 118.59 (14)°.

The water mol­ecule is involved in hydrogen bonds with N1⋯H3*A* = 1.96 (2) Å and O3⋯H6 = 2.548 (9) Å, where the corresponding angles are O3—H3*A*⋯N1 = 178 (15)° and C6—H6⋯O3 = 148.6 (3)°, respectively. Hydrogen-bond geometries for (**1**) are summarized in Table 1 ▸.

**Figure 2:**
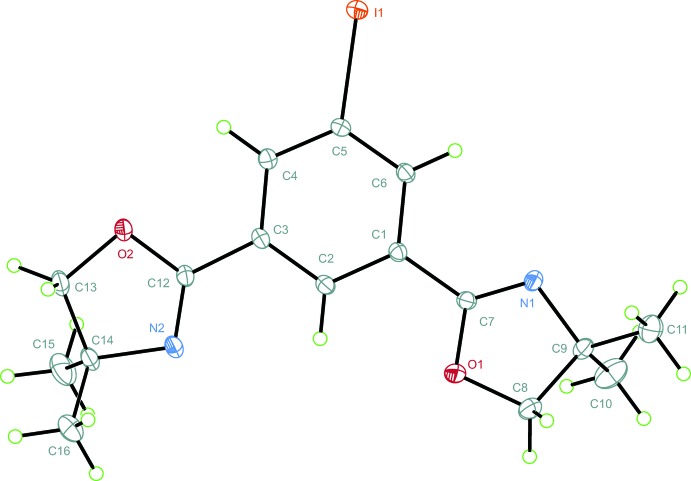
The asymmetric unit of (**2**), shown with displacement ellipsoids drawn at the 50% probability level. H atoms are drawn as green spheres of arbitrary radius.

The isostructural iodo derivative (**2**) was crystallized from di­chloro­methane with no evidence of co-crystallized solvent. Again, an anti­periplanar orientation of the 2-oxazolinyl moieties within the mol­ecular structure (Fig. 2 ▸) was found. In contrast to (**1**), a more distinct twisting of the planes N1/C7/O1 and N2/C12/O2 compared to the plane *B* with inter­planar angles of 16.16 (4) and 15.14 (4)°, respectively, was found.

For (**2**), similar conformational considerations as for (**1**) apply. Puckering parameters *τ_m_* = 19.6 (1)°, *q*_2_ = 0.1902 (11) Å, and *φ*_2_ = 135.5 (3)° indicate a conformation between ^C8^ *T*_C9_ and ^C8^ *E* for ring *C*. A higher degree of planarity was found for ring *A* with *τ_m_* = 4.6 (1)°. The bond length C5—I1 is 2.0968 (9) Å with I1 located 0.1183 (14) Å above ring *B* and thus considerably more distant than the bromine atom in (**1**), where Br1 lies only 0.005 (2) Å above plane *B*. The deviation occurs away from a π–π stacked (see also below) inversion-equivalent formula unit and might be the consequence of steric repulsion between the bulky iodine substituent and the 2-oxazolinyl groups of the neighboring formula unit. Noteworthy deviations from ideal angles within ring *B* were found with C4—C5—C6 = 121.35 (9)° and C3—C4—C5 = 118.60 (9)°. I1 shows an angular adjustment to C6—C5—I1 = 117.08 (7)°, thus improving inter­molecular N⋯I contacts (see below).

All bond lengths for (**1**) and (**2**) fall within expected ranges (Allen *et al.*, 1987 ▸).

## Supra­molecular features   

**Figure 3:**
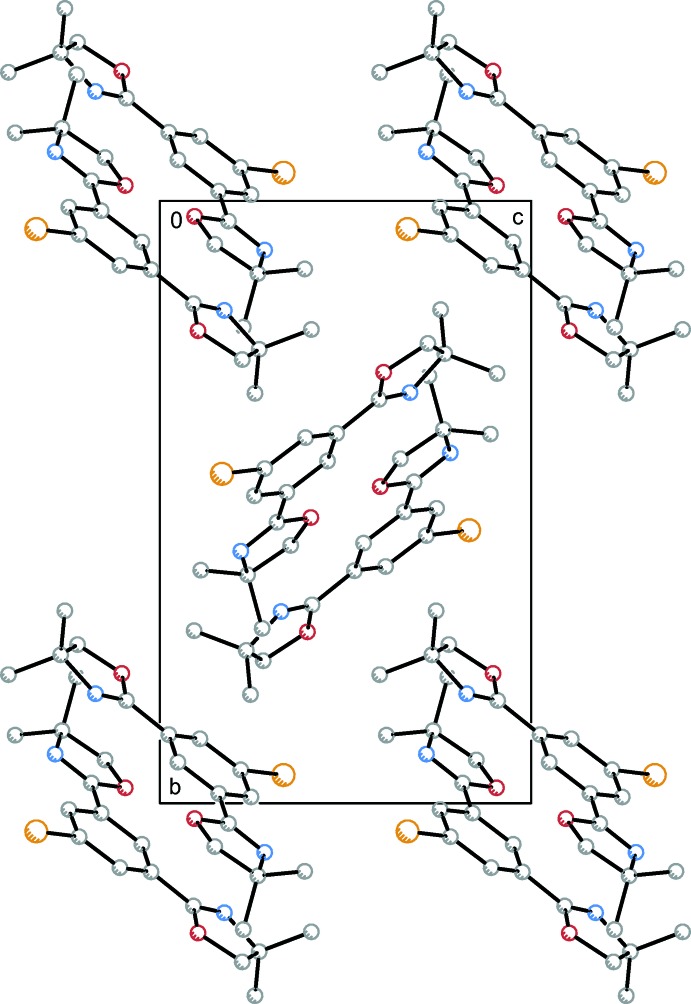
View along the *a* axis of the crystal packing of compound (**1**).

Within the crystal structure of (**1**) (Fig. 3 ▸), inter­molecular bonding is established by classical and non-classical hydrogen bonding (see Table 1 ▸) as well as π–π inter­actions. Close contacts O3⋯H16*B* ^i^ = 2.181 (10) Å with C16^i^—H16*B* ^i^⋯O3 = 146.2 (3)° and H13*B*⋯O3^i^ = 2.936 (11) Å with C13—H13*B*⋯O3^i^ = 130.4 (3)° were found. Further hydrogen-bonding inter­actions arise from mutual inter­actions of two inversion-related water mol­ecules with H3*B*⋯O3^iii^ = 2.32 (13) Å and O3—H3*B*⋯O3^iii^ = 126 (13)°.

**Figure 4:**
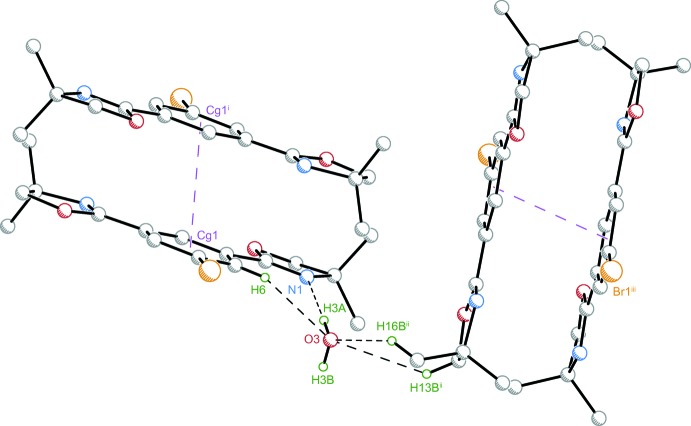
Inter­molecular contacts within the crystal structure of (**1**) are established by means of parallel-displaced π–π inter­actions and hydrogen bonding. Displacement ellipsoids are at the 50% probability level and inter­molecular contacts are depicted as dashed lines. Only H atoms involved in hydrogen bonding or van der Waals contacts (black dashed lines) are shown as green spheres at an arbitrary radius. Purple dashed lines indicate centroid–centroid connecting lines. [Symmetry codes: (i) −*x* + 1, −*y* + 1, −*z* + 1; (ii) −*x* + 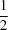, *y* + 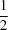, −*z* + 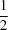; (iii) *x* − 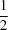, −*y* + 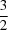, *z* − 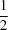.]

Additionally, parallel-displaced π–π stacking with *Cg*1⋯*Cg*1^i^ = 3.5064 (12) Å (*Cg*1 denotes the centroid calculated for the arene atoms) and a horizontal displacement of *I* = 0.996 (2) Å (for a description of geometric parameters of π–π associated arenes, see: Snyder *et al.*, 2012 ▸) between the centroids was found. The inter­planar distance is *R* = 3.3620 (13) Å. The π–π inter­action gives rise to anti­periplanar dimers linked to each other by an inversion. Features of inter­molecular bonding for (**1**) are illustrated in Fig. 4 ▸.

**Figure 5:**
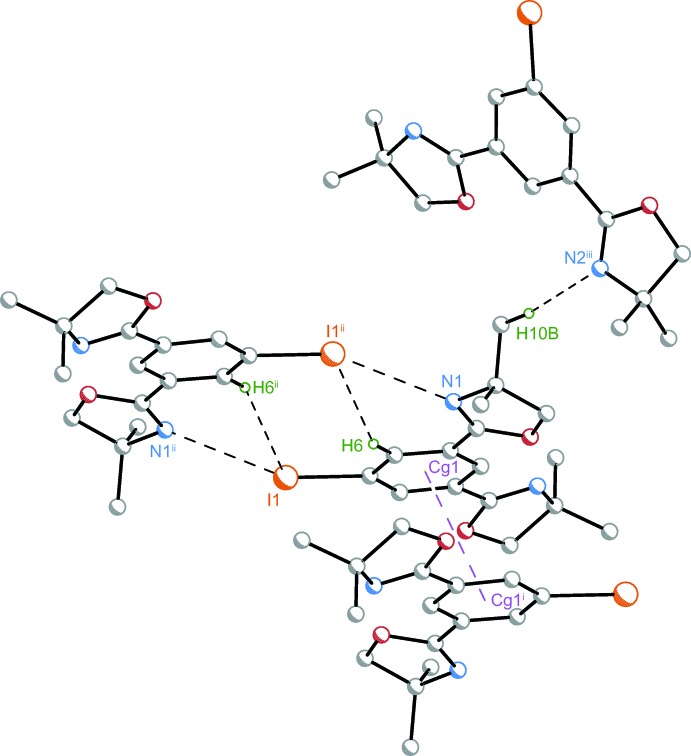
Supra­molecular features within the crystal structure of (**2**) comprise π–π contacts and mutual N1⋯I1^ii^ and N1^ii^⋯I1 inter­actions. Moreover, there are non-conventional hydrogen bonds I1^ii^⋯H6, I1⋯H6^ii^, and H10*B*⋯N2^iii^. Inter­molecular contacts are shown as dashed lines. Only atoms H6, H6^ii^, and H10*B* which are involved in hydrogen bonding (black dashed lines) are shown as green spheres of an arbitrary radius. Purple dashed lines connect centroids of π–π associated dimers. [Symmetry codes: (i) −*x* + 1, −*y* + 1, −*z* + 1; (ii) −*x* + 1, −*y* + 2, −*z* + 1; (iii) −*x* + 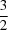, *y* + 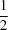, −*z* + 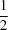.]

**Figure 6:**
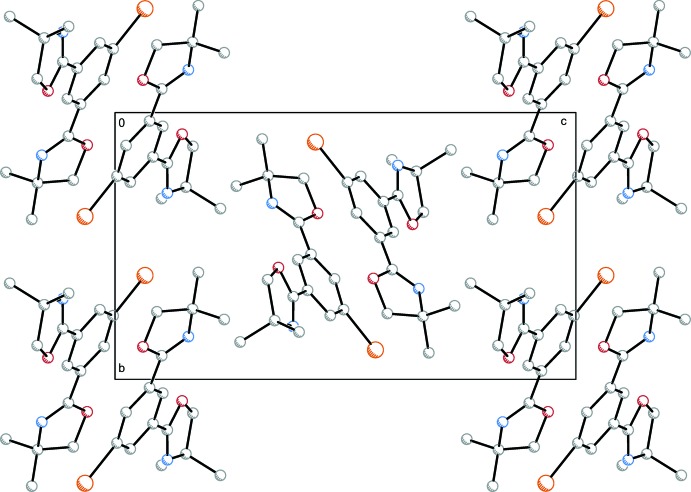
View along the *a* axis of the crystal packing of compound (**2**).

The supra­molecular contacts found for (**2**) are depicted in Fig. 5 ▸. A comparable π–π stacking motif as for compound (**1**) was found within the crystal structure of (**2**) (Fig. 6 ▸). The centroid–centroid distance *Cg*1⋯*Cg*1^i^ = 3.6142 (8) Å is slightly increased compared to (**1**). The inter­planar distance is *R* = 3.2862 (9) Å with a remarkably increased horizontal centroid–centroid displacement of *I* = 1.5044 (15) Å.

Mutual hydrogen bonds I1⋯H6^ii^ = I1^ii^⋯H6 = 3.11 Å with C6—H6⋯I1^ii^ = C6^ii^—H6^ii^⋯I1 = 150° and H10*B*⋯N2^ii^ = 2.745 Å with an angle of C10—H10*B*⋯N2^ii^ = 172° establish inter­molecular bonding (Table 2 ▸). Additionally, particularly short mutual N1⋯I1^ii^ and N1^ii^⋯I1 contacts at 3.2779 (9) Å were found. The N⋯I distance corresponds to 89% of the sum of the van der Waals radii (3.70 Å; Alvarez, 2013 ▸) of the atoms involved. To the best of our knowledge, the shortest N⋯I contact found in a crystal structure was recently reported to be 2.622 Å (Bosch, 2014 ▸), corresponding to 71% of the sum of the van der Waals radii. With respect to I1⋯N1^ii^ contacts, the angle C5—I1⋯N1^ii^ = 155.61 (3)° is found to be slightly linearized by an angular adaption C6—C5—I1 (see above).

The halogen-halogen distances for (**1**) and (**2**) exceed the sum of the van der Waals radii and lack appropriate angles for halogen–halogen bonding.

## Database survey   

The crystal structure of the reagent used to prepare compounds (**1**) and (**2**) (see also below), 5-bromo-1,3-di­cyano­benzene, has been published recently (CCDC reference number DIRLEX; Seidel *et al.*, 2013 ▸).

A WebCSD search (Version 1.1.1, updated July 2015; Groom & Allen, 2014 ▸) for the 1,3-bis­(1,3-oxazolin-2-yl)benzene substructure gave 143 hits. Very few of the crystal structures show the parent motif without any metal ion bound to it. The following considerations take into account only purely organic structures with a 1,3-substitutional pattern, although a significant number of (metal coordinated) 1,3,5-tri(1,3-oxazolin-2-yl)benzenes has been reported.

1-Iodo-2,6-bis­(4′-isopropyl-1,3-oxazolin-2-yl)-4-*tert*-butylbenzene (ROHMIL; Bugarin & Connell, 2008 ▸) and 1,3-bis­(4,4-dimethyl-1,3-oxazolin-2-yl)-2-(tri­methyl­stann­yl)benzene (FILNAQ; Stol *et al.*, 2005 ▸) show substitutional variation from compounds (**1**) and (**2**). By introducing sterically demanding substituents *ortho* to the 1,3-oxazolin-2-yl groups, these are considerably more twisted against the parent arene ring plane with twisting angles ranging from 47.3 (2)–63.6 (2)° and 22.35 (8)–22.75 (8)° for the iodo and tin derivatives, respectively. The more distinct twisting of the respective groups in (**2**) with 16.16 (4) and 15.14 (4)° compared to (**1**) [2.15 (12) and 3.67 (16)°] might be attributed to geometrical requirements for the formation of supra­molecular bonding observed for (**2**). 1-Iodo-2,6-bis­(4′-isopropyl-1,3-oxazolin-2-yl)-4-*tert*-butyl­benzene shows short N⋯I distances at 3.041 (6) Å corresponding to 82% of the sum of van der Waals radii.

Other structures generally lack halide substitution at the central arene, although substitutional variety at the 4′-positions was found. Instead of 4,4-dimethyl substitution, derivatives bearing hydrogen atoms (LAFNEM; Chen *et al.*, 2004 ▸), hy­droxy­methyl (MODQIH; Javadi *et al.*, 2014 ▸) or isopropyl groups (DOWGOM; Mei *et al.*, 2009 ▸) have been found. For those structures, no features of inter­molecular π–π inter­actions could be observed. Instead, C—H⋯π inter­action becomes obvious for LAFNEM and DOWGOM. It is supposed that halide substitution increases dispersion inter­action at modest horizontal centroid-centroid separations (Arnstein & Sherrill, 2008 ▸), thus promoting π–π stacking for (**1**) and (**2**). As a result of the presence of electronegative atoms, hydrogen bonding and van der Waals contacts can also be found for the structures mentioned.

Structures DOWGOM, FILNAQ, MODQIH, and ROHMIL show a synperiplanar orientation of the 2-oxazolinyl groups. The anti­periplanar arrangement as in (**1**) and (**2**) was only found for LAFNEM.

Langer *et al.* found the same ^C8^ *T*_C9_ conformation (with respect to the numbering scheme used herein) within the 2-oxazoline ring of 2-(4-hy­droxy­phen­yl)-4,4-dimethyl-2-oxazoline (CELMAI; Langer *et al.*, 2006 ▸) as for the 2-oxazoline moieties in (**1**) and (**2**). Conformational analysis of the 2-oxazolines within the structures discussed herein showed that all possible conformations twisted around C8—C9 can be found, that are ^C8^ *T*_C9_ (two times within DOWGOM, CELMAI) and ^C9^ *T*_C8_ (LAWCAO, ROHMIL). Folding into an envelope conformation was only observed at C8 with examples for ^C8^ *E* (two times within DOWGOM, ROHMIL) and *E*_C8_ (FILNAQ, two times within ROHMIL) conformations. This might suggest that folding happens preferentially at the less-substituted methyl­ene position. Planar conformations have been found for MODQIH, LAFNEM, and FILNAQ.

## Synthesis and crystallization   

**Figure 7:**
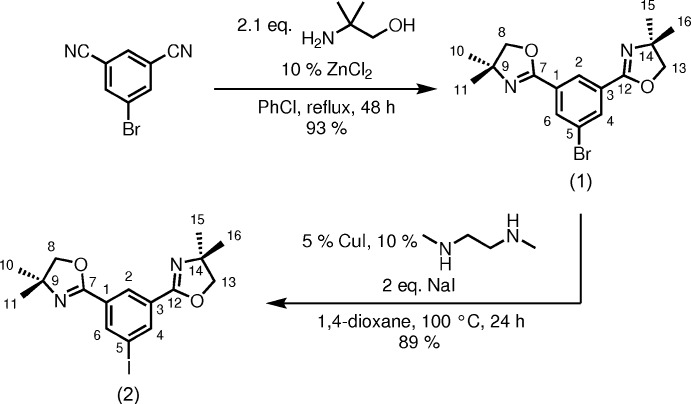
The synthesis scheme of (**1**) and (**2**), starting from 1-bromo-3,5-di­cyano­benzene. The numbering scheme for the title compounds is given.

(**1**) and (**2**) were synthesized starting from 5-bromo-1,3-di­cyano­benzene (Fig. 7 ▸). The multi-step preparation of 5-bromo-1,3-di­cyano­benzene starting from isophthalic acid has been described in a patent (Dillard *et al.*, 2010 ▸). For the preparation of compound (**1**), 5-bromo-1,3-di­cyano­benzene was subjected to cyclization at the cyano positions with 2-amino-2-methyl­propan-1-ol under zinc(II) catalysis (Button *et al.*, 2002 ▸) to give the *meta*-bis­(1,3-oxazolin-2-yl) arene. The iodo derivative (**2**) was synthesized from (**1**) by an aromatic Finkelstein reaction (Klapars & Buchwald, 2002 ▸). All reactions were carried out under an atmosphere of dry nitro­gen using standard Schlenk techniques.

### 1-Bromo-3,5-bis­(4,4-dimethyl-1,3-oxazolin-2-yl)benzene sub-hydrate (**1**)   

Zinc(II) chloride (73 mg, 0.54 mmol, 0.1 eq.) was melted three times *in vacuo* with help of a heat gun. 5-Bromo-1,3-di­cyano­benzene (1.13 g, 4.65 mmol, 1.0 eq.), 2-amino-2-methyl­propan-1-ol (870 mg, 9.77 mmol, 2.1 eq.) and chloro­benzene (15 mL) were added, the colorless suspension was magnetically stirred and heated to reflux for 48 h where it became a pink-colored solution. After qu­anti­tative conversion of the di­cyano compound was confirmed *via* TLC, the reaction mixture was cooled to 353 K. Volatiles were removed *in vacuo* at 353–433 K. After cooling to room temperature, di­chloro­methane (50 mL) and distilled water (50 mL) were added. The organic layer was separated, the aqueous layer extracted with di­chloro­methane (three times with 20 mL each). The combined organic layers were dried over sodium sulfate, filtered, and the solvent was removed from the filtrate under reduced pressure. Column chromatography of the crude product (SiO_2_; petrol ether/ethyl acetate = 4:1) gave (**1**) (1.52 g, 4.33 mmol, 93%) as a colorless solid. Crystallization of (**1**) from a concentrated solution in aceto­nitrile at 277 K gave single crystals suitable for X-ray structural analysis.

^1^H-NMR (DMSO-*d*_6_, 300.21 MHz): *δ* (p.p.m.) = 8.28 (*t*, 1H; ^4^ *J*(H,H) = 1.5 Hz, H2), 8.06 (*d*, 2H; ^4^ *J*(H,H) = 1.5 Hz, H4, H6), 4.16 (*s*, 4H; H8, H13), 1.30 (*s*, 12H; H10, H11, H15, H16). ^13^C{^1^H}-NMR (DMSO-*d*_6_, 75.50 MHz): *δ* (p.p.m.) = 158.7 (C7, C12), 132.5 (C4, C6), 130.1 (C1, C3), 126.0 (C2), 121.9 (C5), 78.9 (C8, C13), 67.7 (C9, C14), 28.1 (C10, C11, C15, C16). ESI–HRMS(+) *m*/*z*: calculated for [*M*+H]^+^ 351.0703/353.0688, found 351.0707/353.0689 (^79^Br/^81^Br).

### 1-Iodo-3,5-bis­(4,4-dimethyl-1,3-oxazolin-2-yl)benzene (2)   

A *J. Young* ampoule was charged with (**1**) (500 mg, 1.42 mmol, 1.0 eq.), copper(I) iodide (14 mg, 74 µmol, 5 mol%), and sodium iodide (426 mg, 2.84 mmol, 2.0 eq.). The ampoule was put to vacuum und flushed with nitro­gen three times. 1,4-Dioxane (3 mL) and *N,N′*-di­methyl­ethylenedi­amine (15 µL, 0.14 mmol, 10 mol%) were added. The ampoule was sealed with a Teflon screw valve, the colorless suspension was magnetically stirred and heated to 373 K for 24 h. An intense blue coloring was observed during the reaction course. After cooling to room temperature, ammonia solution (10 mL, 25%), distilled water (20 mL), and di­chloro­methane (30 mL) were added. The organic layer was separated, the aqueous layer extracted with di­chloro­methane (three times with 10 mL each) and the combined organic phases were dried over sodium sulfate. After filtration, di­chloro­methane was removed from the filtrate under reduced pressure. The crude product was purified by means of column chromatography (SiO_2_; petrol ether/ethyl acetate = 4:1) to give a colorless solid (**2**) (505 mg, 1.27 mmol, 89%). Crystallization from a concentrated solution of (**2**) in di­chloro­methane by slow evaporation of the solvent yielded single crystals suitable for X-ray single crystal diffraction.

^1^H-NMR (DMSO-d_6_, 300.21 MHz): *δ* (p.p.m.) = 8.28 (*t*, 1H; ^4^ *J*(H,H) = 1.5 Hz, H2), 8.24 (*d*, 2H; ^4^ *J*(H,H) = 1.5 Hz, H4, H6), 4.14 (*s*, 4H; H8, H13), 1.29 (*s*, 12H; H10, H11, H15, H16). ^13^C{^1^H}-NMR (DMSO-*d*_6_, 75.50 MHz): *δ* (p.p.m.) = 158.6 (C7, C12), 138.2 (C1, C3), 132.5 (C4, C6), 129.8 (C2), 126.2 (C1), 78.8 (C8, C13), 67.7 (C9, C14), 28.1 (C10, C11, C15, C16). EI–MS *m*/*z*: calcd. for *M* ^+.^ 398.08, found 398.08 (*M* ^+.^), 383.06 (*M* ^+.^ – CH_3_^.^), 368.07 (*M* ^+.^ – 2CH_3_^.^).

## Refinement   

Crystal data, data collection, and structure refinement details are summarized in Table 3 ▸.

Primary atom site locations were assigned with *EDMA* (Palatinus *et al.*, 2012 ▸) from electron densities obtained by *SUPERFLIP* (Palatinus & Chapuis, 2007 ▸). The remaining secondary non-carbon atom sites were located from the difference Fourier map. All non-hydrogen atoms were refined with anisotropic displacement parameters.

Full-matrix least-squares refinement on *F* ^2^ was done with *SHELXL2014/7* (Sheldrick, 2008 ▸). Carbon-bound hydrogen atoms were positioned geometrically and refined riding on their respective carbon atom. Bond lengths were fixed at 0.95 Å (aromatic H), 0.98 Å (methyl H), and 0.99 Å (methyl­ene H). *U*_iso_(H) was fixed at 1.5 *U*_eq_(O) and *U*_eq_(C) for hydroxyl and methyl hydrogens or 1.2 *U*_eq_(C) for the remaining hydrogens. Methyl hydrogens were fitted to the experimental electron density by allowing them to rotate around the C—C bond with a fixed angle (HFIX 137).

For (**1**), occupancy refinement of O3 gave an occupancy of 0.15543 (611) which was subsequently fixed at 0.15. The hydrogen atoms H3*A* and H3*B* were located with the help of CALC-OH, which is the *WinGX* (Farrugia, 2012 ▸) implementation of Nardelli’s method (Nardelli, 1999 ▸) of OH atom positioning. The coordinates of those hydrogen atoms were refined freely while applying restraints to the overall water geometry (O—H = 0.84 Å and H⋯H = 1.328 Å).

## Supplementary Material

Crystal structure: contains datablock(s) 1, 2, global. DOI: 10.1107/S2056989015016059/lh5783sup1.cif

Structure factors: contains datablock(s) 1. DOI: 10.1107/S2056989015016059/lh57831sup2.hkl

Supporting information file. DOI: 10.1107/S2056989015016059/lh57831sup4.cdx

Structure factors: contains datablock(s) 2. DOI: 10.1107/S2056989015016059/lh57832sup3.hkl

Supporting information file. DOI: 10.1107/S2056989015016059/lh57832sup5.cdx

Supporting information file. DOI: 10.1107/S2056989015016059/lh57831sup6.cml

Supporting information file. DOI: 10.1107/S2056989015016059/lh57832sup7.cml

CCDC references: 1420839, 1420838

Additional supporting information:  crystallographic information; 3D view; checkCIF report

## Figures and Tables

**Table 1:** Hydrogen-bond geometry (Å, °) for (**1**)

*D*—H⋯*A*	*D*—H	H⋯*A*	*D*⋯*A*	*D*—H⋯*A*
C6—H6⋯O3	0.95	2.55 (1)	3.395 (10)	149 (1)
O3—H3*A*⋯N1	0.84 (2)	1.96 (2)	2.796 (10)	178 (15)
C16—H16*B*⋯O3^i^	0.98	2.18 (1)	3.045 (11)	146 (1)
C13—H13*B*⋯O3^i^	0.99	2.94 (1)	3.656 (12)	130 (1)
C8—H8*B*⋯Br1^ii^	0.99	3.09 (1)	4.054 (2)	165 (1)
C11—H11*C*⋯O3^iii^	0.98	2.56 (1)	3.391 (12)	143 (1)
O3—H3*B*⋯N1^iii^	0.84 (2)	2.37 (9)	3.107 (11)	148 (15)
O3—H3*B*⋯O3^iii^	0.84 (2)	2.32 (13)	2.90 (2)	126 (13)

Symmetry codes: (i) 
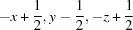
; (ii) 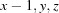; (iii) 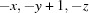.

**Table 2:** Hydrogen-bond geometry (Å, °) for (**2**)

*D*—H⋯*A*	*D*—H	H⋯*A*	*D*⋯*A*	*D*—H⋯*A*
C6—H6⋯I1^i^	0.95	3.11	3.9679 (9)	150
C10—H10*B*⋯N2^ii^	0.98	2.75	3.7228 (15)	172

Symmetry codes: (i) 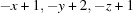; (ii) 
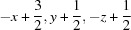
.

**Table 3:** Experimental details

	(**1**)	(**2**)
Crystal data		
Chemical formula	C_16_H_19_BrN_2_O_2_·0.15H_2_O	C_16_H_19_IN_2_O_2_
*M*_r_	353.94	398.23
Crystal system, space group	Monoclinic, *P*2_1_/*n*	Monoclinic, *P*2_1_/*n*
Temperature (K)	100	100
*a*, *b*, *c* (Å)	10.0661 (1), 16.2960 (2), 11.0400 (1)	9.6195 (2), 9.9759 (2), 17.2951 (4)
β (°)	114.496 (2)	94.648 (1)
*V* (Å^3^)	1647.96 (4)	1654.23 (6)
*Z*	4	4
Radiation type	Mo *K*α	Mo *K*α
μ (mm^−1^)	2.50	1.94
Crystal size (mm)	0.34 × 0.26 × 0.08	0.22 × 0.12 × 0.08

Data collection		
Diffractometer	Agilent SuperNova Dual Source diffractometer with an Atlas detector	Bruker *SMART* *APEX* CCD area-detector diffractometer
Absorption correction	Multi-scan (*CrysAlis PRO*; Agilent, 2013 ▸)	Numerical (*SADABS*; Bruker, 2014 ▸)
*T*_min_, *T*_max_	0.670, 1.000	0.649, 0.747
No. of measured, independent and observed [*I* > 2σ(*I*)] reflections	29541, 5438, 4598	45346, 6463, 6035
*R*_int_	0.035	0.019
(sin θ/λ)_max_ (Å^−1^)	0.735	0.776

Refinement		
*R*[*F* ^2^ > 2σ(*F* ^2^)], *wR*(*F* ^2^), *S*	0.035, 0.080, 1.05	0.017, 0.043, 1.07
No. of reflections	5438	6463
No. of parameters	209	194
No. of restraints	3	0
H-atom treatment	H atoms treated by a mixture of independent and constrained refinement	H-atom parameters constrained
Δρ_max_, Δρ_min_ (e Å^−3^)	0.85, −0.74	0.82, −0.62

Computer programs: *CrysAlis PRO* (Agilent, 2013 ▸), *APEX2* and *SAINT* (Bruker, 2014 ▸), *SUPERFLIP* (Palatinus & Chapuis, 2007 ▸), *SHELXL2014* (Sheldrick, 2015 ▸), *XP* in *SHELXTL* (Sheldrick, 2008 ▸), *WinGX* (Farrugia, 2012 ▸), *OLEX2* (Dolomanov *et al.*, 2009 ▸), *publCIF* (Westrip, 2010 ▸) and *PLATON* (Spek, 2009 ▸).

## supplementary crystallographic information

### (1) 1-Bromo-3,5-bis(4,4-dimethyl-1,3-oxazolin-2-yl)benzene 0.15-hydrate Crystal data

**Table:**

C_16_H_19_BrN_2_O_2_·0.15H_2_O	*F*(000) = 726
*M**_r_* = 353.94	*D*_x_ = 1.427 Mg m^−^^3^
Monoclinic, *P*2_1_/*n*	Mo *K*α radiation, λ = 0.71073 Å
*a* = 10.0661 (1) Å	Cell parameters from 11733 reflections
*b* = 16.2960 (2) Å	θ = 3.2–31.8°
*c* = 11.0400 (1) Å	µ = 2.50 mm^−^^1^
β = 114.496 (2)°	*T* = 100 K
*V* = 1647.96 (4) Å^3^	Plate, colorless
*Z* = 4	0.34 × 0.26 × 0.08 mm

### (1) 1-Bromo-3,5-bis(4,4-dimethyl-1,3-oxazolin-2-yl)benzene 0.15-hydrate Data collection

**Table:**

Agilent SuperNova Dual Source diffractometer with an Atlas detector	5438 independent reflections
Radiation source: SuperNova (Mo) X-ray Source	4598 reflections with *I* > 2σ(*I*)
Mirror monochromator	*R*_int_ = 0.035
Detector resolution: 10.4127 pixels mm^-1^	θ_max_ = 31.5°, θ_min_ = 3.2°
ω scans	*h* = −14→14
Absorption correction: multi-scan (*CrysAlis PRO*; Agilent, 2013)	*k* = −23→23
*T*_min_ = 0.670, *T*_max_ = 1.000	*l* = −16→16
29541 measured reflections	

### (1) 1-Bromo-3,5-bis(4,4-dimethyl-1,3-oxazolin-2-yl)benzene 0.15-hydrate Refinement

**Table:**

Refinement on *F*^2^	Primary atom site location: iterative
Least-squares matrix: full	Secondary atom site location: difference Fourier map
*R*[*F*^2^ > 2σ(*F*^2^)] = 0.035	Hydrogen site location: mixed
*wR*(*F*^2^) = 0.080	H atoms treated by a mixture of independent and constrained refinement
*S* = 1.05	*w* = 1/[σ^2^(*F*_o_^2^) + (0.0278*P*)^2^ + 1.5569*P*] where *P* = (*F*_o_^2^ + 2*F*_c_^2^)/3
5438 reflections	(Δ/σ)_max_ = 0.001
209 parameters	Δρ_max_ = 0.85 e Å^−^^3^
3 restraints	Δρ_min_ = −0.74 e Å^−^^3^

### (1) 1-Bromo-3,5-bis(4,4-dimethyl-1,3-oxazolin-2-yl)benzene 0.15-hydrate Special details

**Table:**

Geometry. All e.s.d.'s (except the e.s.d. in the dihedral angle between two l.s. planes) are estimated using the full covariance matrix. The cell e.s.d.'s are taken into account individually in the estimation of e.s.d.'s in distances, angles and torsion angles; correlations between e.s.d.'s in cell parameters are only used when they are defined by crystal symmetry. An approximate (isotropic) treatment of cell e.s.d.'s is used for estimating e.s.d.'s involving l.s. planes.
Refinement. Refinement of *F*^2^ against ALL reflections. The weighted *R*-factor *wR* and goodness of fit *S* are based on *F*^2^, conventional *R*-factors *R* are based on *F*, with *F* set to zero for negative *F*^2^. The threshold expression of *F*^2^ > σ(*F*^2^) is used only for calculating *R*-factors(gt) *etc*. and is not relevant to the choice of reflections for refinement. *R*-factors based on *F*^2^ are statistically about twice as large as those based on *F*, and *R*-factors based on ALL data will be even larger. All non-hydrogen atoms were refined with anisotropic displacement parameters. Carbon-bound hydrogen atoms were positioned geometrically and refined riding on their respective carbon atom. Bond lengths were fixed at 0.95 Å (aromatic H), 0.98 Å (methyl H), and 0.99 Å (methylene H). *U*_iso_(H) was fixed at 1.5 *U*_eq_(O) and *U*_eq_(C) for hydroxyl and methyl H atoms or 1.2 *U*_eq_(C) for the remaining H atoms. Methyl H atoms were fitted to the experimental electron density by allowing them to rotate around the C–C bond with a fixed angle (HFIX 137). Occupancy refinement of O3 gave an occupancy of 0.15543 (611) which was subsequently fixed at 0.15. The H atoms H3A and H3B were located with help of *CALC*-OH which is the *WinGX* (Farrugia, 2012) implementation of Nardelli's method (Nardelli, 1999) of OH atom positioning. The coordinates of those H atoms were refined freely while applying restraints to the overall water geometry (O–H = 0.84 Å and H···H = 1.328 Å).

### (1) 1-Bromo-3,5-bis(4,4-dimethyl-1,3-oxazolin-2-yl)benzene 0.15-hydrate Fractional atomic coordinates and isotropic or equivalent isotropic displacement parameters (Å^2^)

**Table:**

	*x*	*y*	*z*	*U*_iso_*/*U*_eq_	Occ. (<1)
C1	0.26376 (17)	0.48374 (9)	0.34172 (15)	0.0162 (3)	
C3	0.44468 (17)	0.38604 (9)	0.47611 (15)	0.0161 (3)	
C5	0.45798 (18)	0.44445 (10)	0.28229 (15)	0.0180 (3)	
C4	0.51639 (17)	0.39301 (10)	0.39218 (15)	0.0176 (3)	
H4	0.6035	0.3631	0.4099	0.021*	
C12	0.49957 (17)	0.32940 (10)	0.59022 (16)	0.0184 (3)	
C7	0.13236 (18)	0.53354 (9)	0.31540 (15)	0.0172 (3)	
C6	0.33230 (18)	0.48959 (10)	0.25469 (15)	0.0179 (3)	
H6	0.2934	0.5238	0.1783	0.022*	
C9	−0.0526 (2)	0.62058 (12)	0.23624 (19)	0.0268 (4)	
C14	0.53332 (19)	0.25360 (11)	0.76730 (17)	0.0226 (3)	
C16	0.4386 (3)	0.18050 (16)	0.7561 (4)	0.0682 (10)	
H16A	0.4962	0.1380	0.8186	0.102*	
H16B	0.4009	0.1589	0.6651	0.102*	
H16C	0.3568	0.1968	0.7771	0.102*	
C15	0.5965 (4)	0.28651 (17)	0.9079 (2)	0.0632 (9)	
H15A	0.6590	0.2447	0.9687	0.095*	
H15B	0.5170	0.3004	0.9336	0.095*	
H15C	0.6544	0.3357	0.9127	0.095*	
C2	0.31925 (17)	0.43183 (9)	0.45143 (15)	0.0159 (3)	
H2	0.2716	0.4276	0.5096	0.019*	
C13	0.6535 (3)	0.2366 (2)	0.7218 (3)	0.0757 (12)	
H13A	0.7495	0.2531	0.7914	0.091*	
H13B	0.6568	0.1775	0.7028	0.091*	
C8	−0.0606 (2)	0.57193 (12)	0.35229 (19)	0.0257 (4)	
H8A	−0.0676	0.6094	0.4201	0.031*	
H8B	−0.1460	0.5347	0.3203	0.031*	
C10	−0.0128 (3)	0.70964 (14)	0.2754 (3)	0.0525 (7)	
H10A	−0.0925	0.7364	0.2894	0.079*	
H10B	0.0031	0.7380	0.2043	0.079*	
H10C	0.0766	0.7120	0.3578	0.079*	
C11	−0.1925 (3)	0.6130 (2)	0.1101 (2)	0.0564 (8)	
H11A	−0.2755	0.6304	0.1283	0.085*	
H11C	−0.2061	0.5558	0.0801	0.085*	
H11B	−0.1859	0.6479	0.0405	0.085*	
N2	0.44454 (16)	0.31821 (9)	0.67357 (14)	0.0217 (3)	
N1	0.07115 (17)	0.58157 (10)	0.21786 (15)	0.0248 (3)	
O2	0.61878 (15)	0.28455 (9)	0.60266 (13)	0.0309 (3)	
O1	0.07500 (14)	0.52563 (8)	0.40654 (13)	0.0257 (3)	
Br1	0.55330 (2)	0.45321 (2)	0.16706 (2)	0.02642 (6)	
O3	0.0751 (12)	0.5663 (7)	−0.0328 (10)	0.035 (2)	0.15
H3A	0.076 (18)	0.572 (11)	0.043 (8)	0.050*	0.15
H3B	0.010 (15)	0.532 (9)	−0.071 (14)	0.050*	0.15

### (1) 1-Bromo-3,5-bis(4,4-dimethyl-1,3-oxazolin-2-yl)benzene 0.15-hydrate Atomic displacement parameters (Å^2^)

**Table:**

	*U*^11^	*U*^22^	*U*^33^	*U*^12^	*U*^13^	*U*^23^
C1	0.0199 (7)	0.0131 (6)	0.0129 (6)	−0.0033 (5)	0.0042 (5)	−0.0015 (5)
C3	0.0180 (7)	0.0150 (7)	0.0121 (6)	−0.0028 (5)	0.0030 (5)	−0.0007 (5)
C5	0.0211 (7)	0.0192 (7)	0.0136 (6)	−0.0068 (6)	0.0072 (6)	−0.0031 (6)
C4	0.0180 (7)	0.0174 (7)	0.0148 (7)	−0.0036 (5)	0.0042 (6)	−0.0034 (5)
C12	0.0174 (7)	0.0176 (7)	0.0172 (7)	0.0005 (6)	0.0041 (6)	0.0016 (6)
C7	0.0211 (7)	0.0143 (7)	0.0148 (6)	−0.0027 (5)	0.0060 (6)	−0.0007 (5)
C6	0.0227 (7)	0.0157 (7)	0.0123 (6)	−0.0040 (6)	0.0042 (6)	−0.0002 (5)
C9	0.0225 (8)	0.0287 (9)	0.0283 (9)	0.0055 (7)	0.0098 (7)	0.0115 (7)
C14	0.0214 (8)	0.0232 (8)	0.0218 (8)	0.0059 (6)	0.0075 (6)	0.0091 (6)
C16	0.0363 (13)	0.0327 (12)	0.097 (2)	−0.0059 (10)	−0.0107 (13)	0.0361 (14)
C15	0.091 (2)	0.0425 (14)	0.0272 (11)	0.0238 (14)	−0.0041 (13)	0.0066 (10)
C2	0.0195 (7)	0.0135 (6)	0.0131 (6)	−0.0025 (5)	0.0054 (5)	−0.0011 (5)
C13	0.0707 (19)	0.115 (3)	0.0664 (18)	0.071 (2)	0.0532 (16)	0.0711 (19)
C8	0.0246 (8)	0.0265 (8)	0.0263 (9)	0.0063 (7)	0.0108 (7)	0.0070 (7)
C10	0.0556 (15)	0.0216 (10)	0.097 (2)	0.0112 (10)	0.0485 (16)	0.0155 (12)
C11	0.0293 (11)	0.099 (2)	0.0326 (12)	0.0073 (13)	0.0045 (9)	0.0261 (14)
N2	0.0229 (7)	0.0216 (7)	0.0193 (6)	0.0065 (5)	0.0075 (5)	0.0086 (5)
N1	0.0263 (7)	0.0266 (8)	0.0202 (7)	0.0060 (6)	0.0084 (6)	0.0076 (6)
O2	0.0269 (7)	0.0420 (8)	0.0269 (7)	0.0164 (6)	0.0143 (5)	0.0178 (6)
O1	0.0260 (6)	0.0299 (7)	0.0245 (6)	0.0090 (5)	0.0138 (5)	0.0126 (5)
Br1	0.02744 (9)	0.03482 (10)	0.02055 (9)	−0.00288 (7)	0.01352 (7)	0.00215 (7)
O3	0.039 (5)	0.040 (6)	0.024 (4)	−0.002 (4)	0.011 (4)	0.003 (4)

### (1) 1-Bromo-3,5-bis(4,4-dimethyl-1,3-oxazolin-2-yl)benzene 0.15-hydrate Geometric parameters (Å, º)

**Table:**

C1—C2	1.391 (2)	C14—C13	1.515 (3)
C1—C6	1.399 (2)	C16—H16A	0.9800
C1—C7	1.474 (2)	C16—H16B	0.9800
C3—C2	1.393 (2)	C16—H16C	0.9800
C3—C4	1.396 (2)	C15—H15A	0.9800
C3—C12	1.472 (2)	C15—H15B	0.9800
C5—C6	1.384 (2)	C15—H15C	0.9800
C5—C4	1.389 (2)	C2—H2	0.9500
C5—Br1	1.8898 (16)	C13—O2	1.443 (3)
C4—H4	0.9500	C13—H13A	0.9900
C12—N2	1.269 (2)	C13—H13B	0.9900
C12—O2	1.362 (2)	C8—O1	1.454 (2)
C7—N1	1.266 (2)	C8—H8A	0.9900
C7—O1	1.358 (2)	C8—H8B	0.9900
C6—H6	0.9500	C10—H10A	0.9800
C9—N1	1.485 (2)	C10—H10B	0.9800
C9—C10	1.520 (3)	C10—H10C	0.9800
C9—C11	1.520 (3)	C11—H11A	0.9800
C9—C8	1.537 (3)	C11—H11C	0.9800
C14—N2	1.487 (2)	C11—H11B	0.9800
C14—C16	1.498 (3)	O3—H3A	0.84 (2)
C14—C15	1.511 (3)	O3—H3B	0.84 (2)
			
C2—C1—C6	120.34 (15)	C14—C15—H15A	109.5
C2—C1—C7	120.67 (14)	C14—C15—H15B	109.5
C6—C1—C7	118.99 (14)	H15A—C15—H15B	109.5
C2—C3—C4	120.20 (14)	C14—C15—H15C	109.5
C2—C3—C12	119.44 (14)	H15A—C15—H15C	109.5
C4—C3—C12	120.35 (14)	H15B—C15—H15C	109.5
C6—C5—C4	122.04 (15)	C1—C2—C3	120.03 (15)
C6—C5—Br1	118.98 (12)	C1—C2—H2	120.0
C4—C5—Br1	118.98 (13)	C3—C2—H2	120.0
C5—C4—C3	118.75 (15)	O2—C13—C14	106.17 (16)
C5—C4—H4	120.6	O2—C13—H13A	110.5
C3—C4—H4	120.6	C14—C13—H13A	110.5
N2—C12—O2	118.75 (15)	O2—C13—H13B	110.5
N2—C12—C3	126.08 (15)	C14—C13—H13B	110.5
O2—C12—C3	115.16 (14)	H13A—C13—H13B	108.7
N1—C7—O1	118.85 (15)	O1—C8—C9	104.25 (14)
N1—C7—C1	126.06 (15)	O1—C8—H8A	110.9
O1—C7—C1	115.09 (13)	C9—C8—H8A	110.9
C5—C6—C1	118.59 (14)	O1—C8—H8B	110.9
C5—C6—H6	120.7	C9—C8—H8B	110.9
C1—C6—H6	120.7	H8A—C8—H8B	108.9
N1—C9—C10	108.11 (16)	C9—C10—H10A	109.5
N1—C9—C11	110.53 (19)	C9—C10—H10B	109.5
C10—C9—C11	111.9 (2)	H10A—C10—H10B	109.5
N1—C9—C8	103.30 (14)	C9—C10—H10C	109.5
C10—C9—C8	110.73 (19)	H10A—C10—H10C	109.5
C11—C9—C8	111.89 (17)	H10B—C10—H10C	109.5
N2—C14—C16	109.10 (15)	C9—C11—H11A	109.5
N2—C14—C15	109.82 (16)	C9—C11—H11C	109.5
C16—C14—C15	110.5 (2)	H11A—C11—H11C	109.5
N2—C14—C13	103.34 (15)	C9—C11—H11B	109.5
C16—C14—C13	113.3 (3)	H11A—C11—H11B	109.5
C15—C14—C13	110.6 (2)	H11C—C11—H11B	109.5
C14—C16—H16A	109.5	C12—N2—C14	106.82 (14)
C14—C16—H16B	109.5	C7—N1—C9	106.76 (15)
H16A—C16—H16B	109.5	C12—O2—C13	104.63 (15)
C14—C16—H16C	109.5	C7—O1—C8	105.11 (13)
H16A—C16—H16C	109.5	H3A—O3—H3B	105 (5)
H16B—C16—H16C	109.5		

### (1) 1-Bromo-3,5-bis(4,4-dimethyl-1,3-oxazolin-2-yl)benzene 0.15-hydrate Hydrogen-bond geometry (Å, º)

**Table:**

*D*—H···*A*	*D*—H	H···*A*	*D*···*A*	*D*—H···*A*
C6—H6···O3	0.95	2.55 (1)	3.395 (10)	149 (1)
O3—H3*A*···N1	0.84 (2)	1.96 (2)	2.796 (10)	178 (15)
C16—H16*B*···O3^i^	0.98	2.18 (1)	3.045 (11)	146 (1)
C13—H13*B*···O3^i^	0.99	2.94 (1)	3.656 (12)	130 (1)
C8—H8*B*···Br1^ii^	0.99	3.09 (1)	4.054 (2)	165 (1)
C11—H11*C*···O3^iii^	0.98	2.56 (1)	3.391 (12)	143 (1)
O3—H3*B*···N1^iii^	0.84 (2)	2.37 (9)	3.107 (11)	148 (15)
O3—H3*B*···O3^iii^	0.84 (2)	2.32 (13)	2.90 (2)	126 (13)

Symmetry codes: (i) −*x*+1/2, *y*−1/2, −*z*+1/2; (ii) *x*−1, *y*, *z*; (iii) −*x*, −*y*+1, −*z*.

### (2) 3,5-Bis(4,4-dimethyl-1,3-oxazolin-2-yl)-1-iodobenzene Crystal data

**Table:**

C_16_H_19_IN_2_O_2_	*F*(000) = 792
*M**_r_* = 398.23	*D*_x_ = 1.599 Mg m^−^^3^
Monoclinic, *P*2_1_/*n*	Mo *K*α radiation, λ = 0.71073 Å
*a* = 9.6195 (2) Å	Cell parameters from 9629 reflections
*b* = 9.9759 (2) Å	θ = 2.3–34.2°
*c* = 17.2951 (4) Å	µ = 1.94 mm^−^^1^
β = 94.648 (1)°	*T* = 100 K
*V* = 1654.23 (6) Å^3^	Block, colorless
*Z* = 4	0.22 × 0.12 × 0.08 mm

### (2) 3,5-Bis(4,4-dimethyl-1,3-oxazolin-2-yl)-1-iodobenzene Data collection

**Table:**

Bruker SMART APEX CCD area-detector diffractometer	6463 independent reflections
Radiation source: Incoatec microfocus	6035 reflections with *I* > 2σ(*I*)
Mirror monochromator	*R*_int_ = 0.019
ω scans	θ_max_ = 33.5°, θ_min_ = 2.4°
Absorption correction: numerical (*SADABS*; Bruker, 2014)	*h* = −14→14
*T*_min_ = 0.649, *T*_max_ = 0.747	*k* = −15→15
45346 measured reflections	*l* = −26→26

### (2) 3,5-Bis(4,4-dimethyl-1,3-oxazolin-2-yl)-1-iodobenzene Refinement

**Table:**

Refinement on *F*^2^	Primary atom site location: iterative
Least-squares matrix: full	Secondary atom site location: difference Fourier map
*R*[*F*^2^ > 2σ(*F*^2^)] = 0.017	Hydrogen site location: inferred from neighbouring sites
*wR*(*F*^2^) = 0.043	H-atom parameters constrained
*S* = 1.07	*w* = 1/[σ^2^(*F*_o_^2^) + (0.0189*P*)^2^ + 0.8336*P*] where *P* = (*F*_o_^2^ + 2*F*_c_^2^)/3
6463 reflections	(Δ/σ)_max_ = 0.007
194 parameters	Δρ_max_ = 0.82 e Å^−^^3^
0 restraints	Δρ_min_ = −0.62 e Å^−^^3^

### (2) 3,5-Bis(4,4-dimethyl-1,3-oxazolin-2-yl)-1-iodobenzene Special details

**Table:**

Geometry. All e.s.d.'s (except the e.s.d. in the dihedral angle between two l.s. planes) are estimated using the full covariance matrix. The cell e.s.d.'s are taken into account individually in the estimation of e.s.d.'s in distances, angles and torsion angles; correlations between e.s.d.'s in cell parameters are only used when they are defined by crystal symmetry. An approximate (isotropic) treatment of cell e.s.d.'s is used for estimating e.s.d.'s involving l.s. planes.
Refinement. Refinement of *F*^2^ against ALL reflections. The weighted *R*-factor *wR* and goodness of fit *S* are based on *F*^2^, conventional *R*-factors *R* are based on *F*, with *F* set to zero for negative *F*^2^. The threshold expression of *F*^2^ > σ(*F*^2^) is used only for calculating *R*-factors(gt) *etc*. and is not relevant to the choice of reflections for refinement. *R*-factors based on *F*^2^ are statistically about twice as large as those based on *F*, and *R*-factors based on ALL data will be even larger. All non-hydrogen atoms were refined with anisotropic displacement parameters. Carbon-bound hydrogen atoms were positioned geometrically and refined riding on their respective carbon atom. Bond lengths were fixed at 0.95 Å (aromatic H), 0.98 Å (methyl H), and 0.99 Å (methylene H). *U*_iso_(H) was fixed at 1.5 *U*_eq_(O) and *U*_eq_(C) for hydroxyl and methyl H atoms or 1.2 *U*_eq_(C) for the remaining H atoms. Methyl H atoms were fitted to the experimental electron density by allowing them to rotate around the C–C bond with a fixed angle (HFIX 137).

### (2) 3,5-Bis(4,4-dimethyl-1,3-oxazolin-2-yl)-1-iodobenzene Fractional atomic coordinates and isotropic or equivalent isotropic displacement parameters (Å^2^)

**Table:**

	*x*	*y*	*z*	*U*_iso_*/*U*_eq_	
C1	0.56410 (10)	0.66623 (9)	0.41768 (5)	0.01116 (15)	
C2	0.48728 (10)	0.55181 (9)	0.39654 (5)	0.01198 (15)	
H2	0.5232	0.4878	0.3627	0.014*	
C3	0.35754 (10)	0.53146 (9)	0.42507 (5)	0.01135 (15)	
C4	0.30480 (10)	0.62363 (9)	0.47619 (6)	0.01244 (16)	
H4	0.2179	0.6083	0.4971	0.015*	
C5	0.38251 (10)	0.73850 (9)	0.49576 (6)	0.01210 (15)	
C6	0.51110 (10)	0.76077 (9)	0.46704 (6)	0.01225 (15)	
H6	0.5626	0.8397	0.4808	0.015*	
C7	0.70012 (10)	0.69002 (9)	0.38670 (5)	0.01184 (15)	
C8	0.89158 (12)	0.62676 (11)	0.33104 (8)	0.0221 (2)	
H8A	0.8999	0.6078	0.2754	0.027*	
H8B	0.9694	0.5822	0.3621	0.027*	
C9	0.89278 (10)	0.77946 (10)	0.34634 (6)	0.01420 (16)	
C10	0.88285 (14)	0.85997 (15)	0.27141 (7)	0.0273 (2)	
H10A	0.8013	0.8304	0.2381	0.041*	
H10B	0.9674	0.8460	0.2444	0.041*	
H10C	0.8735	0.9554	0.2834	0.041*	
C11	1.01869 (12)	0.82249 (13)	0.39920 (7)	0.0236 (2)	
H11A	1.0107	0.9179	0.4116	0.035*	
H11B	1.1038	0.8074	0.3729	0.035*	
H11C	1.0227	0.7699	0.4472	0.035*	
C12	0.27785 (10)	0.41157 (10)	0.39882 (6)	0.01243 (16)	
C13	0.12395 (12)	0.24676 (11)	0.40919 (6)	0.0201 (2)	
H13A	0.0211	0.2409	0.4004	0.024*	
H13B	0.1575	0.1747	0.4454	0.024*	
C14	0.19275 (12)	0.23620 (11)	0.33195 (6)	0.01761 (18)	
C15	0.09128 (15)	0.27569 (14)	0.26324 (7)	0.0281 (3)	
H15A	0.0521	0.3643	0.2727	0.042*	
H15B	0.0158	0.2097	0.2570	0.042*	
H15C	0.1408	0.2784	0.2159	0.042*	
C16	0.25716 (16)	0.09954 (12)	0.31923 (9)	0.0299 (3)	
H16A	0.3090	0.1023	0.2728	0.045*	
H16B	0.1832	0.0320	0.3124	0.045*	
H16C	0.3208	0.0762	0.3643	0.045*	
N1	0.76407 (9)	0.80152 (9)	0.38642 (5)	0.01435 (15)	
N2	0.30375 (10)	0.33974 (9)	0.34100 (5)	0.01682 (16)	
O1	0.75825 (9)	0.58108 (8)	0.35446 (5)	0.01984 (15)	
O2	0.16674 (9)	0.37812 (8)	0.43947 (5)	0.01763 (15)	
I1	0.30572 (2)	0.88951 (2)	0.56511 (2)	0.01650 (2)	

### (2) 3,5-Bis(4,4-dimethyl-1,3-oxazolin-2-yl)-1-iodobenzene Atomic displacement parameters (Å^2^)

**Table:**

	*U*^11^	*U*^22^	*U*^33^	*U*^12^	*U*^13^	*U*^23^
C1	0.0122 (4)	0.0101 (4)	0.0111 (4)	−0.0011 (3)	0.0008 (3)	0.0002 (3)
C2	0.0144 (4)	0.0106 (4)	0.0109 (4)	−0.0012 (3)	0.0007 (3)	−0.0008 (3)
C3	0.0130 (4)	0.0099 (4)	0.0108 (4)	−0.0018 (3)	−0.0007 (3)	0.0001 (3)
C4	0.0125 (4)	0.0113 (4)	0.0134 (4)	−0.0008 (3)	0.0006 (3)	0.0002 (3)
C5	0.0132 (4)	0.0102 (4)	0.0130 (4)	0.0003 (3)	0.0016 (3)	−0.0017 (3)
C6	0.0134 (4)	0.0101 (4)	0.0132 (4)	−0.0011 (3)	0.0008 (3)	−0.0012 (3)
C7	0.0133 (4)	0.0111 (4)	0.0113 (4)	0.0000 (3)	0.0017 (3)	−0.0010 (3)
C8	0.0184 (5)	0.0177 (5)	0.0320 (6)	−0.0007 (4)	0.0120 (4)	−0.0047 (4)
C9	0.0128 (4)	0.0151 (4)	0.0152 (4)	0.0001 (3)	0.0040 (3)	0.0016 (3)
C10	0.0277 (6)	0.0344 (6)	0.0208 (5)	0.0057 (5)	0.0073 (4)	0.0111 (5)
C11	0.0150 (5)	0.0285 (6)	0.0271 (5)	−0.0008 (4)	0.0000 (4)	−0.0028 (4)
C12	0.0129 (4)	0.0119 (4)	0.0124 (4)	−0.0030 (3)	0.0004 (3)	0.0004 (3)
C13	0.0224 (5)	0.0194 (5)	0.0191 (5)	−0.0115 (4)	0.0043 (4)	−0.0044 (4)
C14	0.0203 (5)	0.0153 (4)	0.0174 (4)	−0.0078 (4)	0.0028 (4)	−0.0047 (3)
C15	0.0329 (6)	0.0294 (6)	0.0207 (5)	−0.0104 (5)	−0.0061 (5)	−0.0043 (4)
C16	0.0319 (7)	0.0168 (5)	0.0419 (7)	−0.0062 (4)	0.0090 (6)	−0.0096 (5)
N1	0.0127 (3)	0.0128 (3)	0.0183 (4)	−0.0013 (3)	0.0051 (3)	−0.0004 (3)
N2	0.0191 (4)	0.0150 (4)	0.0168 (4)	−0.0070 (3)	0.0038 (3)	−0.0042 (3)
O1	0.0188 (4)	0.0137 (3)	0.0284 (4)	−0.0021 (3)	0.0107 (3)	−0.0070 (3)
O2	0.0175 (3)	0.0182 (4)	0.0178 (3)	−0.0083 (3)	0.0057 (3)	−0.0050 (3)
I1	0.01488 (3)	0.01529 (3)	0.01976 (4)	−0.00079 (2)	0.00401 (2)	−0.00607 (2)

### (2) 3,5-Bis(4,4-dimethyl-1,3-oxazolin-2-yl)-1-iodobenzene Geometric parameters (Å, º)

**Table:**

C1—C2	1.3924 (13)	C10—H10A	0.9800
C1—C6	1.3959 (13)	C10—H10B	0.9800
C1—C7	1.4728 (13)	C10—H10C	0.9800
C2—C3	1.3936 (13)	C11—H11A	0.9800
C2—H2	0.9500	C11—H11B	0.9800
C3—C4	1.3990 (13)	C11—H11C	0.9800
C3—C12	1.4724 (13)	C12—N2	1.2711 (13)
C4—C5	1.3948 (13)	C12—O2	1.3675 (12)
C4—H4	0.9500	C13—O2	1.4581 (13)
C5—C6	1.3878 (13)	C13—C14	1.5418 (15)
C5—I1	2.0968 (9)	C13—H13A	0.9900
C6—H6	0.9500	C13—H13B	0.9900
C7—N1	1.2713 (12)	C14—N2	1.4847 (13)
C7—O1	1.3620 (12)	C14—C16	1.5209 (17)
C8—O1	1.4494 (14)	C14—C15	1.5271 (17)
C8—C9	1.5460 (15)	C15—H15A	0.9800
C8—H8A	0.9900	C15—H15B	0.9800
C8—H8B	0.9900	C15—H15C	0.9800
C9—N1	1.4835 (13)	C16—H16A	0.9800
C9—C11	1.5191 (15)	C16—H16B	0.9800
C9—C10	1.5209 (15)	C16—H16C	0.9800
			
C2—C1—C6	120.14 (9)	H10B—C10—H10C	109.5
C2—C1—C7	120.32 (8)	C9—C11—H11A	109.5
C6—C1—C7	119.53 (8)	C9—C11—H11B	109.5
C1—C2—C3	119.86 (9)	H11A—C11—H11B	109.5
C1—C2—H2	120.1	C9—C11—H11C	109.5
C3—C2—H2	120.1	H11A—C11—H11C	109.5
C2—C3—C4	120.60 (9)	H11B—C11—H11C	109.5
C2—C3—C12	117.90 (8)	N2—C12—O2	118.56 (9)
C4—C3—C12	121.49 (9)	N2—C12—C3	124.76 (9)
C5—C4—C3	118.60 (9)	O2—C12—C3	116.67 (8)
C5—C4—H4	120.7	O2—C13—C14	104.13 (8)
C3—C4—H4	120.7	O2—C13—H13A	110.9
C6—C5—C4	121.35 (9)	C14—C13—H13A	110.9
C6—C5—I1	117.08 (7)	O2—C13—H13B	110.9
C4—C5—I1	121.49 (7)	C14—C13—H13B	110.9
C5—C6—C1	119.41 (9)	H13A—C13—H13B	108.9
C5—C6—H6	120.3	N2—C14—C16	109.93 (10)
C1—C6—H6	120.3	N2—C14—C15	108.15 (9)
N1—C7—O1	118.80 (9)	C16—C14—C15	111.15 (10)
N1—C7—C1	125.93 (9)	N2—C14—C13	102.51 (8)
O1—C7—C1	115.26 (8)	C16—C14—C13	113.27 (10)
O1—C8—C9	104.86 (8)	C15—C14—C13	111.38 (10)
O1—C8—H8A	110.8	C14—C15—H15A	109.5
C9—C8—H8A	110.8	C14—C15—H15B	109.5
O1—C8—H8B	110.8	H15A—C15—H15B	109.5
C9—C8—H8B	110.8	C14—C15—H15C	109.5
H8A—C8—H8B	108.9	H15A—C15—H15C	109.5
N1—C9—C11	109.38 (9)	H15B—C15—H15C	109.5
N1—C9—C10	108.82 (9)	C14—C16—H16A	109.5
C11—C9—C10	110.82 (10)	C14—C16—H16B	109.5
N1—C9—C8	103.37 (8)	H16A—C16—H16B	109.5
C11—C9—C8	112.08 (9)	C14—C16—H16C	109.5
C10—C9—C8	112.05 (10)	H16A—C16—H16C	109.5
C9—C10—H10A	109.5	H16B—C16—H16C	109.5
C9—C10—H10B	109.5	C7—N1—C9	107.12 (8)
H10A—C10—H10B	109.5	C12—N2—C14	106.86 (9)
C9—C10—H10C	109.5	C7—O1—C8	105.40 (8)
H10A—C10—H10C	109.5	C12—O2—C13	104.14 (8)
			
C6—C1—C2—C3	−0.31 (14)	C2—C3—C12—O2	166.31 (9)
C7—C1—C2—C3	−178.83 (9)	C4—C3—C12—O2	−14.56 (14)
C1—C2—C3—C4	−1.37 (14)	O2—C13—C14—N2	18.91 (11)
C1—C2—C3—C12	177.77 (9)	O2—C13—C14—C16	137.30 (10)
C2—C3—C4—C5	2.25 (14)	O2—C13—C14—C15	−96.53 (10)
C12—C3—C4—C5	−176.86 (9)	O1—C7—N1—C9	1.19 (13)
C3—C4—C5—C6	−1.49 (14)	C1—C7—N1—C9	−177.34 (9)
C3—C4—C5—I1	175.31 (7)	C11—C9—N1—C7	−124.42 (10)
C4—C5—C6—C1	−0.15 (14)	C10—C9—N1—C7	114.38 (10)
I1—C5—C6—C1	−177.09 (7)	C8—C9—N1—C7	−4.88 (11)
C2—C1—C6—C5	1.07 (14)	O2—C12—N2—C14	2.75 (13)
C7—C1—C6—C5	179.60 (9)	C3—C12—N2—C14	−175.98 (9)
C2—C1—C7—N1	162.51 (10)	C16—C14—N2—C12	−134.30 (11)
C6—C1—C7—N1	−16.02 (15)	C15—C14—N2—C12	104.17 (11)
C2—C1—C7—O1	−16.07 (13)	C13—C14—N2—C12	−13.58 (12)
C6—C1—C7—O1	165.40 (9)	N1—C7—O1—C8	3.37 (13)
O1—C8—C9—N1	6.63 (11)	C1—C7—O1—C8	−177.95 (9)
O1—C8—C9—C11	124.30 (10)	C9—C8—O1—C7	−6.05 (12)
O1—C8—C9—C10	−110.37 (11)	N2—C12—O2—C13	10.14 (13)
C2—C3—C12—N2	−14.94 (15)	C3—C12—O2—C13	−171.02 (9)
C4—C3—C12—N2	164.19 (10)	C14—C13—O2—C12	−17.51 (11)

### (2) 3,5-Bis(4,4-dimethyl-1,3-oxazolin-2-yl)-1-iodobenzene Hydrogen-bond geometry (Å, º)

**Table:**

*D*—H···*A*	*D*—H	H···*A*	*D*···*A*	*D*—H···*A*
C6—H6···I1^i^	0.95	3.11	3.9679 (9)	150
C10—H10*B*···N2^ii^	0.98	2.75	3.7228 (15)	172

Symmetry codes: (i) −*x*+1, −*y*+2, −*z*+1; (ii) −*x*+3/2, *y*+1/2, −*z*+1/2.
